# Prevalence, Awareness, Treatment, and Control of Hypertension among Adult Residents of Tehran: The Tehran Cohort Study

**DOI:** 10.5334/gh.1120

**Published:** 2022-05-11

**Authors:** Alireza Oraii, Akbar Shafiee, Arash Jalali, Farshid Alaeddini, Soheil Saadat, Saeed Sadeghian, Hamidreza Poorhosseini, Mohamamdali Boroumand, Abbasali Karimi, Oscar H. Franco

**Affiliations:** 1 Tehran Heart Center, Cardiovascular Diseases Research Institute, Tehran University of Medical Sciences, Tehran, Iran; 2 Research Center for Health Management in Mass Gathering, Red Crescent Society of the Islamic Republic of Iran, Tehran, Iran; 3 Department of Emergency Medicine, University of California, Irvine, California, USA; 4 Institute of Social and Preventive Medicine (ISPM), University of Bern, Bern, Switzerland

**Keywords:** Hypertension, Blood pressure, Prevalence, Awareness, Control, Epidemiology

## Abstract

**Background:**

High levels of blood pressure (BP) remain undetected and poorly controlled in large segments of the population leading to an enormous burden in terms of disease and mortality.

**Objective:**

We aimed to assess the prevalence, awareness, treatment, and control of hypertension in Tehran.

**Methods:**

We used the data of 8,296 adults aged ≥35 years from the Tehran Cohort Study who were enrolled between May 2016 and February 2019. Hypertension was defined as systolic BP ≥140 mmHg and/or diastolic BP ≥90 mmHg, self-report, and/or current antihypertensive medication use. The age- and sex-weighted prevalence of hypertension and high normal BP was calculated using the 2016 national census. Furthermore, awareness, treatment, and control of hypertension were analyzed.

**Results:**

The mean age of the participants was 53.8 ±12.75 years, and 54.0% were women. The weighted prevalence of hypertension and high normal BP were 36.5% and 12.2%, respectively. Among hypertensive individuals, 68.2% were aware of hypertension, 53.3% were receiving medication, and 40.4% had adequate BP control. The awareness, treatment, and control of hypertension were significantly higher in women (72.2% vs. 63.4% [P < 0.001], 55.1% vs 51.1% [P = 0.020], and 42.7% vs. 37.7% [P = 0.004], respectively) and this gap considerably increased with advancing age. Hypertension was more prevalent in northern Tehran but with a better treatment rate and control in the same regions.

**Conclusion:**

Despite the high prevalence of hypertension in the adult population of Tehran, the rates of awareness, treatment, and control of hypertension are unsatisfactory and demand comprehensive strategies to improve this situation, especially in younger men.

## Introduction

Hypertension is the most common modifiable cardiovascular disease (CVD) risk factor, contributing to 13% of all-cause global mortality and nearly half of CVD-related and cerebrovascular-related deaths [1,2]. The global burden and prevalence of hypertension have experienced an increasing trend in the past decade due to the aging population, changes in lifestyle and behavioral risk factors, and lack of physical activity, especially in developing countries [3]. Moreover, the global rates of awareness, treatment, and control of hypertension are considerably low, with large disparities among different countries and socioeconomic groups [4].

Tehran, the capital city of Iran, is the third-largest metropolitan area in the Middle East, inhabiting a large population of urban and rural immigrants from different country regions. Epidemiological studies investigating the prevalence of hypertension in Tehran are limited by small sample size, diagnosis of hypertension solely based on participants’ self-report, district-level sampling, and lack of data regarding awareness, treatment, and control of hypertension [5,6,7]. Furthermore, the current status of hypertension prevalence, awareness, and control rate based on different cut-off levels for the definition of hypertension remains unclear.

Multiple country-level epidemiological studies have evaluated the burden of hypertension among the Iranian population, and regional survey studies have reported varying rates of hypertension in different regions of the country [8,9]. These studies have used the higher cut-off levels proposed by the European Society of Cardiology/European Society of Hypertension (ESC/ESH) to define hypertension. However, there is a growing trend toward using more stringent cut-off levels recommended by the American College of Cardiology/American Heart Association (ACC/AHA) among Iranian physicians. However, data regarding the impact of the used guidelines on the prevalence and treatment of hypertension is lacking.

The burgeoning prevalence of hypertension demands a comprehensive study to provide updated data regarding the epidemiology of hypertension for guiding future health policies. Also, recent urbanizations and the emergence of new definitions for hypertension might have led to changes in the related prevalences. We herewith aimed to investigate the prevalence of hypertension, awareness, treatment, and control rates, as well as their age and sex distribution among a large sample of adults enrolled in the Tehran Cohort study. As part of a secondary analysis, we compared the influence of different definitions of elevated blood pressure (BP) proposed by the 2018 ESC/ESH and 2017 ACC/AHA guidelines on the rates of the mentioned parameters.

## Methods

### Study design and participants

In this study, we used the data from the Tehran Cohort Study (TeCS), an ongoing population-based prospective study of adult residents of Tehran. Details of the study design have been previously published [10]. In brief, a systematic sampling method (a variant of simple random sampling) was used to enroll a representative sample of adults from all districts of Tehran. A total of 4,215 households comprising 9,170 adults aged ≥35 years were invited for an interview, and 8,296 individuals (90.5%) participated in the study between May 2016 and February 2019. For this analysis, participants lacking data on BP measurements or a history of hypertension were excluded (n = 39). In the end, data of 8,257 participants were utilized for further analyses. The protocol of TeCS was approved by the Deputy of Research and the Committee of Ethics at Tehran University of Medical Sciences (IR.TUMS.MEDICINE.REC.1399.074). Written informed consent was obtained from all participants before enrollment.

### Data collection and measurements

The participants were interviewed using comprehensive questionnaires designed to gather information regarding demographic characteristics, socioeconomic status, lifestyle, metabolic, behavioral risk factors, preexisting comorbidities, and medical history. Besides, all individuals underwent standard anthropometric and BP measurements. Fasting blood samples were drawn to measure serum biochemistry, including creatinine levels. Weight and height were measured to calculate the body mass index (BMI) (kg/m^2^). BP was measured using a calibrated digital sphygmomanometer, M6 Comfort Omron (Omron Healthcare, Kyoto, Japan), with an appropriate brachial cuff size. The first reading was recorded from the left arm positioned at the heart level while the patient was seated in a resting position, with the back supported for at least five minutes. If the first reading was above 140/90 mmHg, a second measurement from the same arm was performed after five minutes of resting. The second reading was used for the statistical analyses of participants with two BP measurements.

### Definitions

We used cut-off levels of the 2018 ESC/ESH guideline to define different BP categories [11]. Individuals were labeled as hypertensive if any of the following features were present: 1) systolic BP (SBP) ≥140 mmHg or diastolic BP (DBP) ≥90 mmHg, 2) self-report of a previous hypertension diagnosis by healthcare providers, or 3) current use of any antihypertensive medication. Individuals without a prior diagnosis of hypertension or antihypertensive medication use who had an SBP between 130–140 mmHg and/or DBP between 85–90 mmHg were categorized as having high normal BP. The remainder of the study population not meeting the above criteria were grouped as having normal/optimal BP. Hypertensive participants were further assessed regarding awareness, defined as self-report of a previous diagnosis of hypertension, treatment as self-report of receiving any antihypertensive medication, and controlled BP as SBP <140 mmHg and DBP <90 mmHg. We assessed the impact of different cut-points proposed by the European and American guidelines by performing a secondary analysis to determine the prevalence and rate of BP control using definitions proposed by the 2017 ACC/AHA guideline (hypertension defined as SBP ≥130 mmHg or DBP ≥80 mmHg and elevated BP defined as SBP 120–129 mmHg and DBP <80 mmHg) [12].

Current tobacco use was defined as everyday or occasional cigarette use, pipe, or hookah smoking. Former tobacco users were those who quit for at least one month before the interview. Alcohol consumption was defined as any use of alcoholic products within the preceding year. Opium consumption was defined as any current or previous inhalational or oral use of opium or its derivatives. The prior history of diabetes was described as a self-report of a previous diagnosis of diabetes or the use of glucose-lowering medication and hyperlipidemia as a previous diagnosis of hyperlipidemia or the use of lipid-lowering medication. The amount of daily total physical activity was asked from the patients and categorized as low, intermediate, and high activity based on a Likert-scale questionnaire.

### Statistical analysis

Categorical variables were reported as frequency (percentage) and compared between the study groups using the chi-square test. The age of the participants was expressed as mean with standard deviation (SD). A one-way analysis of variance (ANOVA) test was used for the comparison of age among the normal/optimal, high normal, and hypertension groups. In addition to crude frequencies, age- and sex-weighted prevalence of hypertension and high normal BP were calculated based on Tehran’s adult population ≥35 years in the 2016 national census. Moreover, the weighted prevalence of hypertension and high normal BP in men and women was calculated based on their age distribution. The prevalence of hypertension, awareness, treatment and BP control among various age, sex, BMI, metabolic risk factor, physical activity, and education subgroups were further analyzed to examine variations of mentioned indices by different subpopulations. Finally, the geographic distributions of hypertension, awareness, treatment and control were depicted in the Tehran map based on the first three digits of the participants’ postal code using *shp2dta* and *spmap* modules in Stata software, release 14.2 (College Station, TX: Stata Corp LP.). Statistical analyses were performed using the IBM SPSS Statistics for Windows, version 23 (Armonk, NY: IBM Corp.).

## Results

Data of 8,257 individuals were used for analysis in the present study (99.5% of total TeCS participants). The mean age of the study participants was 53.8 (SD: 12.75) years, and women constituted 54.0% of the participants. The second BP measurement was performed in 1,986 participants. The baseline characteristics of the study population are shown in Table 1.

**Table 1:** Baseline characteristics and prevalence of hypertension in the population of the Tehran Cohort Study.


	TOTAL POPULATION* n = 8296	NORMAL/OPTIMAL^†^ n = 3962 (48.0)	HIGH NORMAL^†^ n = 1007 (12.2)	HYPERTENSION^†^ n = 3288 (39.8)	P-VALUE^‡^

**Age, year, mean (SD)**	53.8 (12.75)	48.60 (10.9)	53.4 (11.77)	60.2 (12.16)	<0.001

**Age, year, n (%)**					<0.001

35–44	2329 (28.1)	1693 (73.1)	262 (11.3)	360 (15.6)	

45–54	2209 (26.6)	1190 (54.1)	312 (14.2)	696 (31.7)	

55–64	1967 (23.7)	722 (36.8)	233 (11.9)	1008 (51.3)	

65–74	1222 (14.7)	265 (21.8)	152 (12.5)	800 (65.7)	

≥75	569 (6.9)	92 (16.3)	48 (8.5)	424 (75.2)	

**Sex, n (%)**					0.013

Men	3818 (46.0)	1794 (47.2)	507 (13.3)	1500 (39.5)	

Women	4478 (54.0)	2168 (48.7)	500 (11.2)	1788 (40.1)	

**Marital status, n (%)**					0.663

Married	8210 (99.2)	3929 (48.0)	1001 (12.2)	3258 (39.8)	

Non-married	86 (0.8)	31 (47.0)	6 (9.1)	29 (43.9)	

**Ethnicity, n (%)**					0.224

Fars	4026 (48.8)	1877 (46.6)	510 (12.7)	1639 (40.7)	

Azari	2447 (29.7)	1187 (48.5)	290 (11.9)	970 (39.6)	

Gilak	330 (4.0)	166 (50.3)	34 (10.3)	130 (39.4)	

Lor	318 (3.9)	175 (55.0)	28 (8.8)	115 (36.2)	

Kurd	193 (2.3)	98 (50.8)	26 (13.5)	69 (35.8)	

Mixed	680 (8.2)	331 (48.7)	79 (11.6)	270 (39.7)	

Other	206 (2.5)	102 (49.5)	32 (15.5)	72 (35.0)	

Immigrants/Refugees	51 (0.6)	23 (45.1)	8 (15.7)	20 (39.2)	

**Education, year, n (%)**					<0.001

Illiterate	585 (7.1)	141 (24.1)	51 (8.7)	393 (67.2)	

(1–5)	841 (10.2)	286 (34.1)	98 (11.7)	455 (54.2)	

(6–12)	4306 (52.0)	2070 (48.2)	559 (13.0)	1662 (38.7)	

>12	2541 (30.7)	1461 (57.6)	299 (11.8)	776 (30.6)	

**Body mass index, kg/m^2^, n (%)**					<0.001

<20	227 (2.8)	158 (69.6)	25 (11.0)	44 (19.4)	

20–24.9	2079 (25.3)	1251 (60.5)	243 (11.7)	575 (27.8)	

25–29.9	3426 (41.7)	1661 (48.6)	438 (12.8)	1320 (38.6)	

30–34.9	1796 (21.9)	680 (37.9)	221 (12.3)	892 (49.7)	

≥35	683 (8.3)	193 (28.3)	76 (11.1)	413 (60.6)	

**Diabetes, n (%)**					<0.001

No	6965 (84.4)	3640 (52.2)	895 (12.8)	2430 (34.9)	

Yes	1292 (15.6)	322 (24.9)	112 (8.7)	858 (66.4)	

**Hyperlipidemia, n (%)**					<0.001

No	5562 (67.4)	3122 (56.1)	759 (13.6)	1681 (30.2)	

Yes	2694 (32.6)	839 (31.1)	248 (9.2)	1607 (59.7)	

**Chronic kidney disease, n (%)**					<0.001

No	8187 (99.1)	3948 (48.2)	1005 (12.3)	3234 (39.5)	

Yes	71 (0.9)	14 (19.7)	2 (2.8)	55 (77.5)	

**Serum Creatinine, mg/dL, median (25^th^, 75^th^ percentile)**	0.80 (0.70, 0.94)	0.79 (0.69, 0.90)	0.80 (0.70, 0.93)	0.84 (0.71, 1.00)	<0.001

**Tobacco user, n (%)**					<0.001

Current	1594 (19.3)	911 (57.2)	185 (11.6)	498 (31.2)	

Former	333 (4.0)	110 (33.0)	40 (12.0)	183 (55.0)	

Never	6325 (76.6)	2937 (46.4)	782 (12.4)	2606 (41.2)	

**Opium consumption, n (%)**					0.309

No	7856 (94.7)	3750 (48.1)	948 (12.2)	3091 (39.7)	

Yes	440 (5.3)	194 (44.4)	58 (13.3)	185 (42.3)	

**Alcohol consumption, n (%)**					0.001

No	7555 (91.0)	3540 (47.3)	919 (12.3)	3024 (40.4)	

Yes	741 (9.0)	402 (54.6)	84 (11.4)	250 (34.0)	

**Physical activity, n (%)**					<0.001

Low	1449 (17.6)	539 (37.3)	127 (8.8)	779 (53.9)	

Intermediate	4764 (58.0)	2311 (48.6)	592 (12.5)	1845 (38.9)	

High	2000 (24.4)	1082 (54.2)	282 (14.1)	632 (31.7)	


* Categorical data are reported as number (percentage in the column). ^†^ Categorical data are reported as number (percentage in a row). ^‡^ P-value is calculated between normal/optimal, high normal, and hypertension subgroups.

### Prevalence

**Figure 1:**
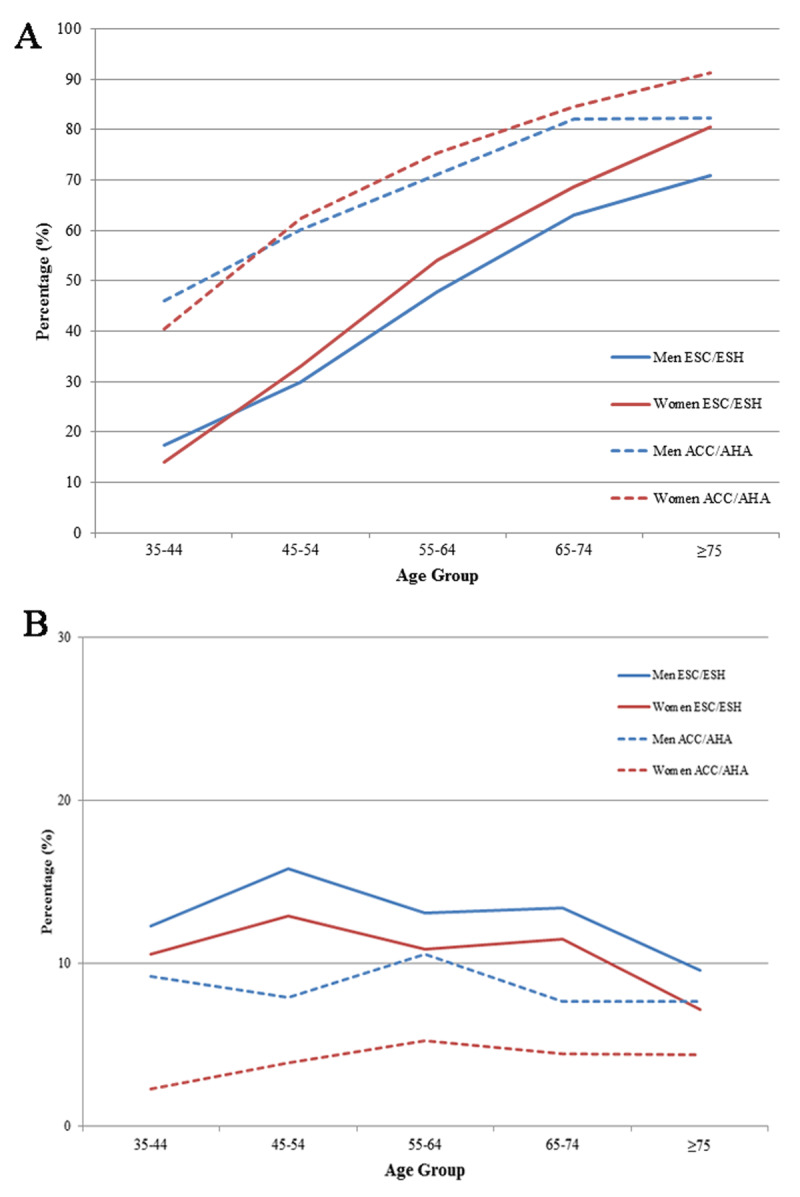
**a)** Prevalence of hypertension stratified by age and sex; **b)** Prevalence of high normal blood pressure stratified by age and sex. (Hypertension cut-off points based on ESC/ESH guideline was ≥140/90 mm Hg and for ACC/AHA guideline was ≥130/80 mm Hg. High-normal blood pressure range based on ESC/ESH guideline was 130–140/85–90 mmHg and for ACC/AHA systolic: 120–129 mmHg and diastolic blood pressure: <80 mmHg).

Based on the 2018 ESC/ESH definitions of hypertension categories described above, the overall prevalence of hypertension was 39.8% in our study participants, and 12.2% of the study population were categorized in the high normal BP group. Furthermore, the weighted prevalence of hypertension and high normal BP were 36.5% (95% CI: 34.5 – 38.5%) and 12.2 % (95% CI: 10.7 – 13.7%), respectively. The prevalence of hypertension was higher in women than men (weighted prevalence: 37.6% [95% CI: 34.9 – 40.3%] vs. 35.4% [95% CI: 32.4 – 38.4%]) and steeply increased with advancing age, reaching a prevalence of 75.2% among individuals over 75 years (Figure 1-a). On the contrary, the prevalence of high normal BP peaked in individuals between 45–54 years, followed by a decreasing trend in the older age categories (Figure 1-b). In contrast with hypertensive individuals, a higher overall prevalence of high normal BP was detected in men than in women (weighted prevalence: 13.3% [95% CI: 11.2 – 15.8%] vs. 11.2% [95% CI: 9.3 – 13.3%]). The prevalence of hypertension was higher in those with higher BMI (≥35: 60.6% vs. <20: 19.4%), preexisting comorbidities (diabetics: 66.4% vs. non-diabetics: 34.9%), and lower physical activity levels (low activity: 53.9% vs. high activity: 31.7%) (Table 1). Moreover, the prevalence of hypertension significantly decreased with more years of education and was nearly two times higher in illiterate individuals than those with ≥12 years of education (illiterate: 67.2% vs. ≥12 years: 30.6%).

**Figure 2:**
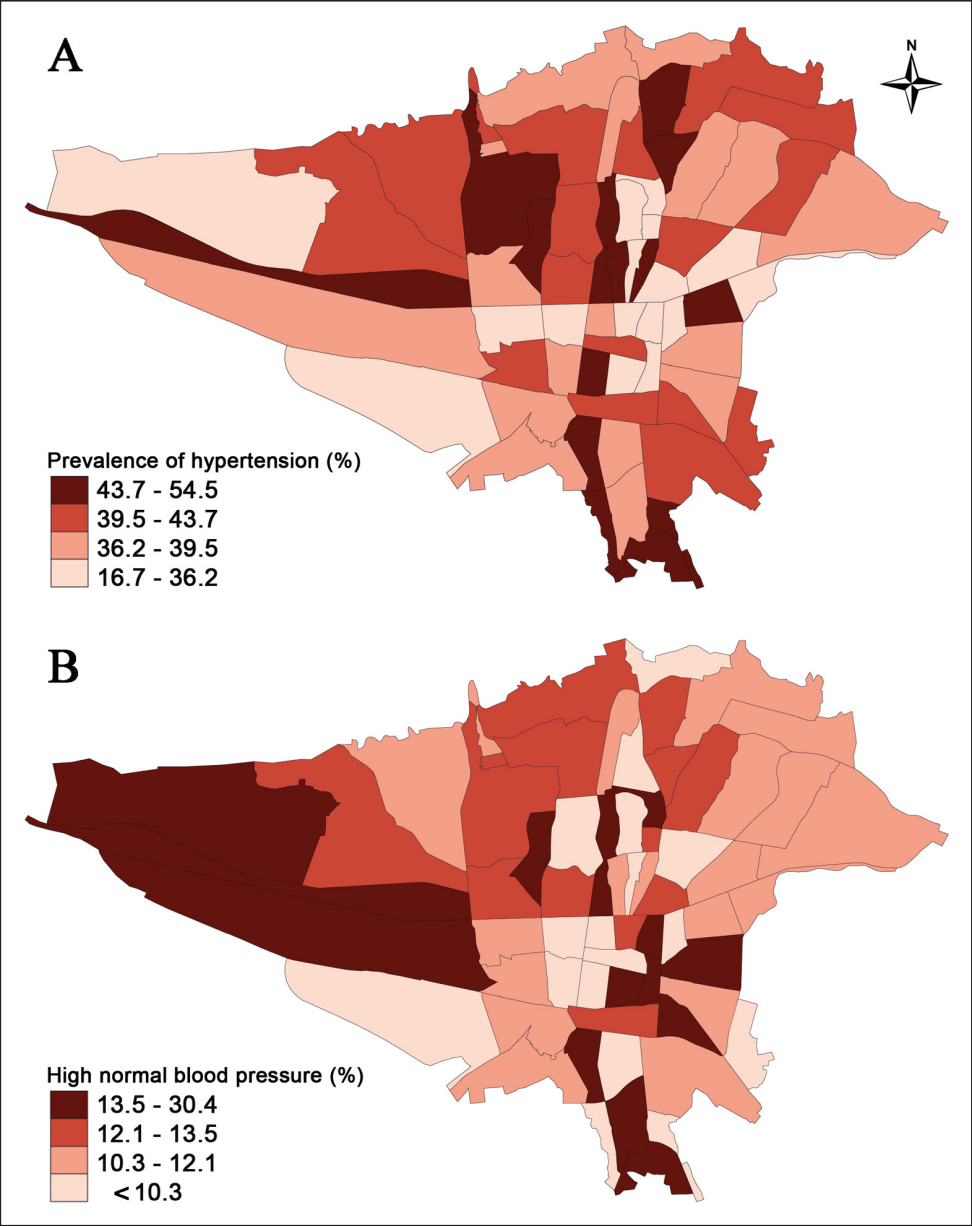
Geographic distribution of **a)** the prevalence of hypertension based on the postal regions of Tehran, **b)** The prevalence of high-normal blood pressure based on the postal regions of Tehran.

As shown in Figure 2-a, the prevalence of hypertension was higher in the mid-northern part of Tehran, but individuals with high-normal blood pressure were more frequently observed in the northwestern quarter of Tehran (Figure 2-b).

### Awareness, treatment, and control

**Figure 3:**
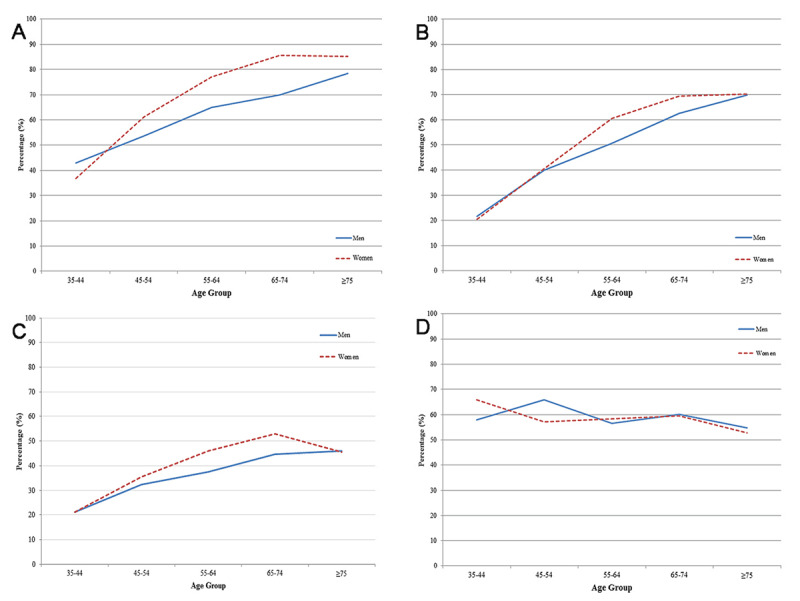
**a)** Awareness among hypertensive individuals stratified by age and sex; **b)** Treatment among hypertensive individuals stratified by age and sex; **c)** Control of blood pressure among hypertensive individuals stratified by age and sex; **d)** Control of blood pressure among hypertensive individuals receiving antihypertensive medication stratified by age and sex.

**Figure 4:**
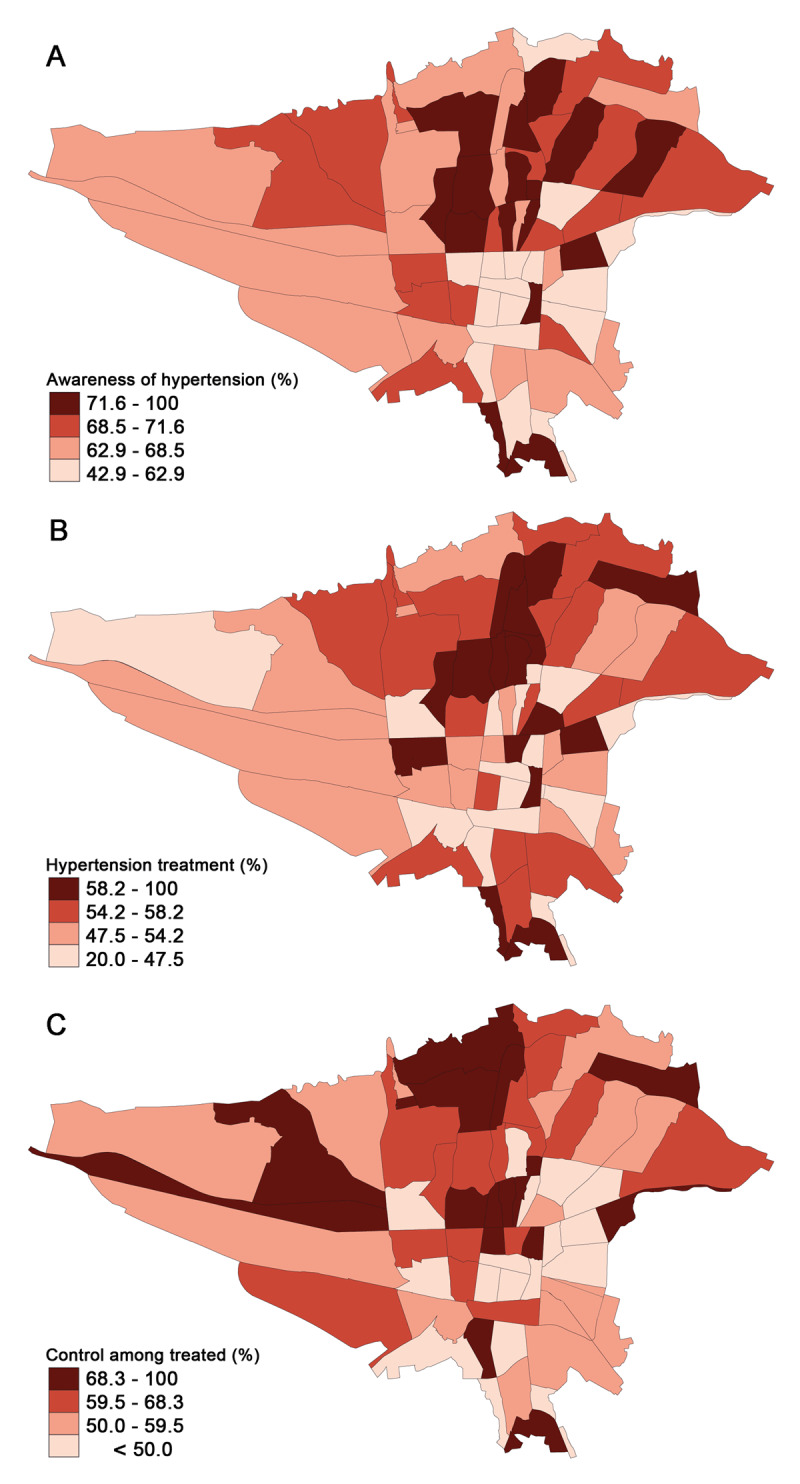
Geographic distribution of **a)** Awareness of hypertension based on the postal regions of Tehran, **b)** Treatment of hypertension based on the postal regions of Tehran, **c)** Control of hypertension among treated patients based on the postal regions of Tehran.

Overall, 68.2% of hypertensive individuals were aware of their hypertension status. Awareness was higher among women than men (72.2% vs. 63.4%) and considerably improved with advancing age (Figure 3-a). The prevalence of awareness was more prominent in the northern half of Tehran (Figure 4-a). Individuals with current tobacco use, alcohol consumption, lower BMI, higher physical activity, and more years of education were significantly less aware of their hypertension status (Table 2).

**Table 2:** Prevalence of awareness, treatment, and control of hypertension in the hypertensive population of the Tehran Cohort Study (n = 3288).


	AWARENESS	TREATMENT	CONTROL	CONTROL AMONG TREATED

**Overall, n (%)**	2242 (68.2)	1752 (53.3)	1328 (40.4)	1023 (58.4)

**Age, year, n (%)**				

35–44	143 (39.7)	76 (21.1)	76 (21.1)	47 (61.8)

45–54	403 (57.9)	281 (40.4)	238 (34.2)	171 (60.9)

55–64	727 (72.1)	569 (56.4)	429 (42.6)	328 (57.6)

65–74	624 (78.0)	529 (66.1)	391 (48.9)	317 (59.9)

≥75	345 (81.6)	297 (70.0)	194 (45.8)	160 (53.9)

p-value	<0.001	<0.001	<0.001	0.378

**Sex, n (%)**				

Men	951 (63.4)	766 (51.1)	565 (37.7)	451 (58.9)

Women	1291 (72.2)	986 (55.1)	763 (42.7)	572 (58.0)

p-value	<0.001	0.020	0.004	0.716

**Marital status, n (%)**				

Married	2224 (68.3)	1737 (53.3)	1316 (40.4)	1013 (58.3)

Non-married	17 (58.6)	15 (51.7)	11 (37.9)	10 (66.7)

p-value	0.266	0.864	0.788	0.514

**Ethnicity, n (%)**				

Fars	1135 (69.3)	906 (55.3)	687 (41.9)	547 (60.4)

Azari	674 (69.5)	522 (53.8)	385 (39.7)	288 (55.2)

Gilak	80 (61.5)	64 (49.2)	52 (40.0)	39 (60.9)

Lor	71 (61.7)	43 (37.4)	40 (34.8)	24 (55.8)

Kurd	49 (71.0)	40 (58.0)	30 (43.5)	26 (65.0)

Mixed	171 (63.3)	125 (46.3)	96 (35.6)	68 (54.4)

Other	48 (66.7)	41 (56.9)	31 (43.1)	25 (61.0)

Immigrants/Refugees	12 (60.0)	9 (45.0)	5 (25.0)	5 (55.6)

P-value	0.166	0.002	0.301	0.580

**Education, year, n (%)**				

Illiterate	315 (80.2)	261 (66.4)	160 (40.7)	129 (49.4)

(1–5)	333 (73.2)	257 (56.5)	163 (35.8)	117 (45.5)

(6–12)	1099 (66.2)	854 (51.4)	678 (40.8)	530 (62.1)

>12	493 (63.5)	379 (48.8)	325 (41.9)	246 (64.9)

P-value	<0.001	<0.001	0.188	<0.001

**Body mass index, kg/m^2^, n (%)**				

<20	27 (61.4)	19 (43.2)	17 (38.6)	10 (52.6)

20–24.9	345 (60.1)	277 (48.2)	230 (40.0)	172 (62.1)

25–29.9	874 (66.2)	692 (52.4)	538 (40.8)	420 (60.7)

30–34.9	656 (73.5)	514 (57.6)	359 (40.2)	282 (54.9)

≥35	304 (73.6)	220 (53.3)	167 (40.4)	122 (55.5)

P-value	<0.001	0.005	0.996	0.150

**Diabetes, n (%)**				

No	1522 (62.6)	1130 (46.5)	880 (36.2)	653 (57.8)

Yes	720 (83.9)	621 (72.4)	447 (52.1)	369 (59.4)

p-value	<0.001	<0.001	<0.001	0.507

**Hyperlipidemia, n (%)**				

No	919 (54.7)	702 (41.8)	473 (28.1)	357 (50.9)

Yes	1323 (82.3)	1049 (65.3)	854 (53.1)	665 (63.4)

P-value	<0.001	<0.001	<0.001	<0.001

**Chronic kidney disease, n (%)**				

No	2193 (67.8)	1710 (52.9)	1296 (40.1)	996 (58.2)

Yes	49 (89.1)	42 (76.4)	32 (58.2)	27 (64.3)

P-value	0.001	0.001	0.007	0.433

**Tobacco user, n (%)**				

Current	300 (60.2)	232 (46.6)	185 (37.1)	146 (62.9)

Former	133 (72.7)	119 (65.0)	85 (46.4)	72 (60.5)

Never	1808 (69.4)	1399 (53.7)	1057 (40.6)	804 (57.5)

P-value	<0.001	<0.001	0.082	0.262

**Opium consumption, n (%)**				

No	2113 (68.4)	1640 (53.1)	1253 (40.5)	958 (58.4)

Yes	121 (65.4)	106 (57.3)	72 (38.9)	63 (59.4)

P-value	0.402	0.262	0.663	0.836

**Alcohol consumption, n (%)**				

No	2085 (68.9)	1626 (53.8)	1230 (40.7)	941 (57.9)

Yes	146 (58.4)	115 (46.0)	87 (34.8)	72 (62.6)

P-value	0.001	0.018	0.069	0.320

**Physical activity, n (%)**				

Low	606 (77.8)	493 (63.3)	342 (43.9)	268 (54.4)

Intermediate	1262 (68.3)	967 (52.3)	737 (39.9)	563 (58.2)

High	357 (56.5)	277 (43.8)	234 (37.0)	179 (64.6)

P-value	<0.001	<0.001	0.028	0.022


Categorical data are reported as numbers (percentage in the row) among individuals with hypertension. P-values are calculated for subgroups in each column independently.

A total of 53.3% of hypertensive individuals were receiving antihypertensive medication at the time of the interview. We observed a higher prevalence of hypertension treatment in the northern half of Tehran (Figure 4-b). This frequency was higher in women than men (55.1% vs. 51.1%), and a higher percentage of individuals received antihypertensive medication with advancing age (Figure 3-b). Also, participants with higher BMI levels were more likely to receive antihypertensive medication (≥35: 53.3% vs. <20: 43.2%); however, higher levels of education and physical activity were inversely associated with receiving antihypertensive medication (Table 2).

Among hypertensive individuals, 40.4% had an adequate level of BP control regardless of receiving medication. Nevertheless, this frequency increased to 58.4% among hypertensive individuals receiving antihypertensive medication. Similar to the geographic treatment pattern, control of hypertension among the treated patients was better in the northern half of Tehran (Figure 4-c). Overall, women had better BP control than men (42.7% vs. 37.7%), while younger participants were more likely to have poor-controlled BP than older individuals (Figure 3-c). However, BP control among treated participants did not vary significantly between age and sex groups (Table 2, Figure 3-d).

### Comparison of the hypertension guidelines

Using the 2017 ACC/AHA guideline definition for hypertension, the prevalence of hypertension increased from 39.8% to 64.0% of the study population (Table 3). Compared with the 2018 ESC/ESH guideline, hypertensive individuals based on the 2017 ACC/AHA guideline had a considerably lower mean age, and a greater proportion of men were classified as hypertensive. Additionally, BP control among hypertensive individuals substantially dropped to 11.1%, four times lower than the control rate based on the 2018 ESC/ESH guideline. Similarly, control of hypertension among participants receiving antihypertensive medication was reduced from 58.4% to 26.7%.

**Table 3:** Prevalence and control of hypertension in the population of the Tehran Cohort Study based on the 2017 ACC/AHA and 2018 ESC/ESH guidelines (n = 8296).


	2018 ESC/ESH	2017 ACC/AHA

Hypertension, n (%)*	3288 (39.8)	5288 (64.0)

Age, mean (SD), year	60.2 (12.16)	56.8 (12.59)

Sex, n (%)		

Men	1500 (39.5)	2447 (64.3)

Women	1788 (40.1)	2841 (63.7)

**Control, n (%)#**	1328 (40.4)	586 (11.1)

Age, mean (SD), year	62.4 (11.09)	63.8 (11.32)

Sex, n (%)		

Men	565 (37.7)	254 (10.4)

Women	763 (42.7)	332 (11.7)

**Control among treated, n (%)^†^**	1023 (58.4)	467 (26.7)

Age, mean (SD), year	63.2 (10.81)	64.4 (11.00)

Sex, n (%)		

Men	451 (58.9)	209 (27.3)

Women	572 (58.0)	258 (26.2)


* Categorical data are reported as numbers (percentage in the row) for each subgroup. ^#^ Percentages calculated from the hypertensive population (n = 3288). ^†^ Percentages calculated from the treated population (n = 1752).

## Discussion

The present study investigated the epidemiology of hypertension using data from the TeCS, which enrolled a representative sample of adult residents of Tehran. The weighted prevalence of hypertension was 36.5% (estimated at about 1.5 million individuals in Tehran metropolis), and high normal BP had a weighted prevalence of 12.2% (estimated at about 500,000 individuals in Tehran metropolis) among the adult population of Tehran aged ≥35 years. Besides, awareness, treatment, and control among treated individuals were 68.2%, 53.3%, and 58.4%, respectively. Despite the higher prevalence of hypertension and high-normal blood pressure in the northern half of Tehran (traditionally with a higher socioeconomic status), better awareness, treatment, and control rates were observed in the same regions. Implementation of the 2017 ACC/AHA guideline for hypertension increased the prevalence of hypertension to as high as 64.0%, reducing the frequency of BP control to 11.1%.

One of the first studies investigating the epidemiology of hypertension in Iran dates back to 1973 in a northern city, which reported that among 550 participants, 30.5% had an SBP > 160 mmHg or DBP > 90 mmHg [13]. Considering the current cut-points, it seems that the prevalence of hypertension was much higher at that time. Since then, multiple national survey studies between 2005 and 2011 showed various prevalence rates of hypertension ranging from 26.9% to 35.4% [14,15,16,17]. Tehran metropolis, a heavily populated and polluted urban city, is possibly one of the regions with a noticeably high prevalence of hypertension in Iran. Moreover, the prevalence of hypertension among adult residents of Tehran has possibly increased over the past years, according to previous studies [6,7]. Air pollution, high salt intake, and physical inactivity among urban dwellers could have aggravated the high prevalence of hypertension in Tehran compared with less industrialized cities and rural areas [18,19,20]. However, a net prevalence rate for hypertension in Tehran was lacking.

In the present study, the prevalence of hypertension increased substantially with advancing age, which is in line with previous findings [21]. We believe the observed pattern of high normal BP and hypertension might be because that individuals between 45–54 years with high normal BP are prone to develop hypertension with increasing age. Healthcare policymakers should implement comprehensive preventive strategies regarding education and early diagnosis of these individuals to decrease the related morbidity and mortality.

We also observed a significant sex difference in the overall prevalence of hypertension and high normal BP. Higher estrogen levels could partially explain the lower prevalence of hypertension among younger women than men, as this pattern does not persist after menopause. At the same time, alternative explanations such as differences in lifestyle or specific hormonal and gynecological characteristics need further exploration [22]. We hypothesize that the sex pattern of hypertension among older individuals could be due to a possible higher survival rate among women than men, as shown in previous studies [23,24], leading to a higher proportion of hypertensive men dying before reaching 75 years. Specific attention should be paid to men between 55–75 years in future educational and screening programs to fill the mentioned gap as these individuals are already at a greater risk of mortality from cardiovascular causes.

The high percentage of unaware hypertensive men and young individuals might be due to employment, lack of time, and less attention to health status in these groups [25]. The lower awareness among men may also be partly explained by the fewer routine health-related visits in men compared to women (e.g., health visits for antenatal care in women). Tobacco use and alcohol consumption were also significantly associated with unawareness and under-treatment. However, individuals with preexisting comorbidities were more likely to be aware, treated, and well-controlled, probably due to more frequent healthcare visits leading to a greater chance of early detection of high BP and initiation of treatment [26]. Accordingly, a study on trends of cardiovascular risk factors showed greater reductions in mean levels of SBP and DBP in patients with diabetes compared with non-diabetics over the past decade [27]. The years of education were inversely correlated with awareness and treatment among hypertensive individuals [28]. Furthermore, although well-educated hypertensive participants were more likely to be unaware of their hypertension status, these individuals had significantly better BP control when medically treated, which points to the possible role of literacy in drug adherence and lifestyle modification. So, health literacy may be a separate topic from the education level, and more social investment is required in this field.

Like the global trend, adequate BP control among Iranian adults has considerably improved over the past years [14]. However, younger individuals had a substantially higher rate of uncontrolled BP in our study. We hypothesize that medication underutilization and poor compliance might be responsible for higher rates of uncontrolled BP in younger individuals. Hence, early initiation of pharmacologic therapy as indicated, single-pill regimens, and telehealth programs should be considered in these individuals to improve drug adherence and BP control [29,30]. A recent systematic review and meta-analysis pooling evidence from 107 national and subnational studies in Iran showed that the BP control rate varies from 4.5% to 68.39% between different geographical regions [31]. This highlights the importance of our region-specific findings among adult residents of Tehran, which is one of the largest metropolitan areas in the Middle East. Policymakers should implement regional data such as ours in their public health strategies for improving awareness, treatment, and control of hypertension.

After using the criteria of the 2017 ACC/AHA guideline with a lower cut-point, the prevalence of hypertension substantially increased from 39.8% to 64.0%. This considerable difference in the prevalence of hypertension based on the mentioned definitions had been previously observed among other populations [32]. However, a recent study among American adults showed that although a higher proportion of the population was labeled as hypertensive, the 2017 ACC/AHA guideline would result in a slight increase in the percentage of individuals requiring antihypertensive medication [33]. The rationale is that BP level and 10-year CVD risk are used in conjunction for decision-making regarding the initiation of antihypertensive medication in the mentioned guideline.

On the contrary, the frequency of individuals with well-controlled BP substantially dropped when the recommended 2017 ACC/AHA definition for BP control was used. The rationale behind the more stringent cut-off for BP control in the 2017 ACC/AHA was derived from the pooled analysis of multiple randomized controlled trials investigating the benefits of more intensive BP control. These studies showed that more intensive BP targets (<120 mmHg vs. <140 mmHg) lead to a risk reduction for fatal and nonfatal major cardiovascular events, myocardial infarction, and stroke [34,35]. At first glance, one would argue about the imposed cost of diagnosis and treatment of individuals based on lower cut-points. Also, stringent BP goals might increase the number of patients requiring multiple antihypertensive medications to reach the recommended target. However, reducing expenses due to the decrease in cardiovascular and cerebrovascular events can justify the initial costs. This was supported in an economic analysis applying SPRINT trial treatment effects of intensive vs. standard BP control strategies [36]. This study showed that despite more healthcare resource utilization, intensive BP control prevents cardiovascular disease events, prolongs longevity, and is cost-effective below common willingness-to-pay thresholds. Hence, cost-benefit analyses and public health assets, such as infrastructure, number of facilities, and availability of medical services, should be considered before incorporating the mentioned new definitions into national health policies, especially in developing and low-income countries.

### Study limitations

Despite being a large-scale epidemiological study investigating hypertension in the adult residents of Tehran from all districts for the first time, our study had some shortcomings. First, we enrolled the adult residents of Tehran aged ≥35 years. So, our findings could not be generalized to the general population as younger individuals were not investigated. Second, BP measurements were performed at a single session, while guidelines recommend confirmation of elevated BP in at least two separate office visits. Although single visit measurements could overestimate the prevalence of hypertension and uncontrolled BP due to white coat hypertension, this has been the most feasible detection method in population-based epidemiological studies. In TeCS, we interviewed participants (nine forms/questionnaires), recorded electrocardiogram, did anthropometric and BP measurements, and performed blood sample testing in a single session. Therefore, we did not measure the second BP reading in individuals with a normal first reading due to time restrictions and limited healthcare resources. We used the described approach to improve the feasibility of performing all the measurements in a single session and increase the willingness among individuals to participate. Therefore, the possibility of some degree of underestimation cannot be completely excluded.

## Conclusion

Levels of high blood pressure are high in Tehran metropolis, with over one-third of its adult population (aged ≥35 years) having hypertension, of whom only 68.2% are aware of their condition. Furthermore, nearly half of hypertensive adult residents of Tehran are receiving antihypertensive medication, and only 40.4% have adequately controlled BP. The awareness, treatment, and control of hypertension were significantly lower among men and younger individuals.

As the first study on a large random sample from the citizens of Tehran, our findings could guide healthcare policymakers in designing and conducting comprehensive preventive strategies to reduce hypertension-related morbidity and mortality further. Furthermore, future studies should focus on these high-risk populations and evaluate the efficacy of different prevention and treatment programs focusing on improving health literacy, medication adherence, and lifestyle behaviors.
